# Naked-Eye Detection of Food-Borne Pathogens Using Multiplex Hyperbranched Rolling Circle Amplification and Magnetic Particles

**DOI:** 10.3390/bios12121075

**Published:** 2022-11-25

**Authors:** Congli Tang, Hongna Liu, Wenjing Pan, Meiling Wang, Jie Ren, Zhu Chen, Hui Chen, Yan Deng, Song Li

**Affiliations:** Hunan Key Laboratory of Biomedical Nanomaterials and Devices, Hunan University of Technology, Zhuzhou 412007, China

**Keywords:** food-borne pathogens, magnetic particles, hyperbranched rolling circle amplification, multiple detection, naked-eye detection

## Abstract

Food safety is a significant public health issue in both developed and developing countries. Previous detection methods struggle to meet the current demands. We have proposed a new way to detect pathogens, allowing detection to be visualized by the naked eye. Using our newly developed assay, when target genes are present in the reaction, corresponding padlock probes form closed-loop molecules. Each reaction tube contains a pair of universal primers for identifying target genes. The ring padlock probes and corresponding universal primers start hyperbranched rolling circle amplification (HRCA) under the action of the polymerase, so as to gain branched chain amplification products, which are irreversibly entangled with magnetic particles to form aggregated magnetic particle clusters, and the detection results are visible to naked eyes. On the contrary, by using linear probes, the clustering of magnetic particles will not be produced. This method was applied to the detection of five food-borne pathogens enterohemorrhagic *Escherichia coli* (EHEC), enterotoxigenic *Escherichia coli* (ETEC), enteropathogenic *Escherichia coli* (EPEC), enteroinvasive *Escherichia coli* (EIEC) and *Escherichia coli* (*E. coli*), with detection limits of 1 × 10^3^, 1 × 10^4^, 1 × 10^3^, 1 × 10^4^ and 1 × 10^2^ CFU/mL, respectively. This method can realize multiplex automatic detection of nucleic acid and shows great development potential in the field of molecular diagnosis.

## 1. Introduction

Food is the most important necessity for the survival of living organisms. Unfortunately, many high-risk pathogens spread through various foods causing human sickness [1]. Food-borne diseases are a major public health matter around the world [2,3]. Pathogens are transmitted through food every day and have become a life-threatening issue affecting millions of people [4], in both developed and developing countries. According to data from the Centers for Disease Control and Prevention, more than 48 million people are ill, 128,000 are hospitalized and 3000 die from food-borne diseases in the United States every year [5]. The potential of a disease outbreak increases as international food trade increases [6]. This is a continuing threat to public health, especially for children, the elderly and pregnant women who have more vulnerable immune systems [7]. Therefore, food safety has become an essential worldwide concern for consumers, industries and regulatory agencies.

In recent years, the simplicity of portable field detection devices and the fact that the detection results are visible to the naked eye have led researchers to study visual detection in depth. Hu et al. established a method for detecting *Staphylococcus aureus* based on recombinase polymerase amplification and polymer flocculation sedimentation [8]. Vidya et al. used gold nanoparticles to detect *E. coli* by colorimetry [9], while Donghoon et al., used platinum-coated magnetic nanoparticle clusters (Pt/MNCs) [10]. In Sang et al., visible detection resulted from a gold enhancement process following the electrostatic interaction between gold nanoparticles and target DNA [11]. Lin et al. proposed a new method for nucleic acid sequence specificity and naked eye detection through isothermal amplification and magnetic particle-mediated aggregation [12]. Although the visualization methods proposed by the above researchers simplify the detection and reduce the dependence on the instrument, these methods can only detect one pathogen at a time, not allowing detection of multiple samples in one experiment.

Therefore, in order to achieve multiplex detection, Mohammadali et al. showed a new type of portable point of care (POC) diagnostic device, which detected both *E. coli* and *Staphylococcus aureus* (*S. aureus*) by colorimetry [13]. Seung-Ho et al. developed and optimized an optical fiber sensor, which can detect three kinds of pathogenic bacteria from food at the same time (*E. coli*, *S. aureus* and *Listeria monocytogenes*) [14]. The principle behind this method is antibody antigen reaction, and the signal needs specific detectors and computers. Shi et al. was also able to simultaneously detect three pathogens (*Salmonella typhimurium*, *S. aureus* and *L. monocytogenes*) in one experiment by using multiplex PCR amplification [15]. Similarly, researchers Maier et al., Li et al., Sun et al. and Zhong et al. adopted multiplex PCR to detect multiple pathogens at once [16,17,18,19]. Suo et al. combined multiple PCR amplifications with microarray technology [20]. A major disadvantage to the Suo et al. method was requiring temperature cycling systems, gel electrophoresis systems and gel imaging systems, which is costly and requires professionally trained personnel. Zhao established a lateral flow detection method based on 10-channel up-conversion fluorescence technology [21]. This method used a laser scanning disk and was able to detect 10 popular food-borne pathogens quickly and simultaneously. Although the above-mentioned methods were able to execute the multiplex detection of pathogens, they are highly dependent on costly instruments and professionally trained operators.

In our research, taking advantage of some excellent properties of functional magnetic nanoparticles (NPs), we will propose a new method of multiple hyperbranched rolling circle amplification based on the naked eye detection of magnetic particles to detect five food-borne pathogens. The method only needs a simple temperature control system to realize constant temperature visual detection of nucleic acid, which is beneficial to the automation and high-throughput detection of nucleic acid.

## 2. Materials and Methods

In this study, using characteristics of functional magnetic particles (MPs), we put forward a new method using multiplex hyperbranched rolling circle amplification (MHRCA-MPND) resulting in naked-eye detection of five food-borne pathogens: *E. coli*, EPEC, ETEC, EHEC and EIEC.

**Figure 1:**
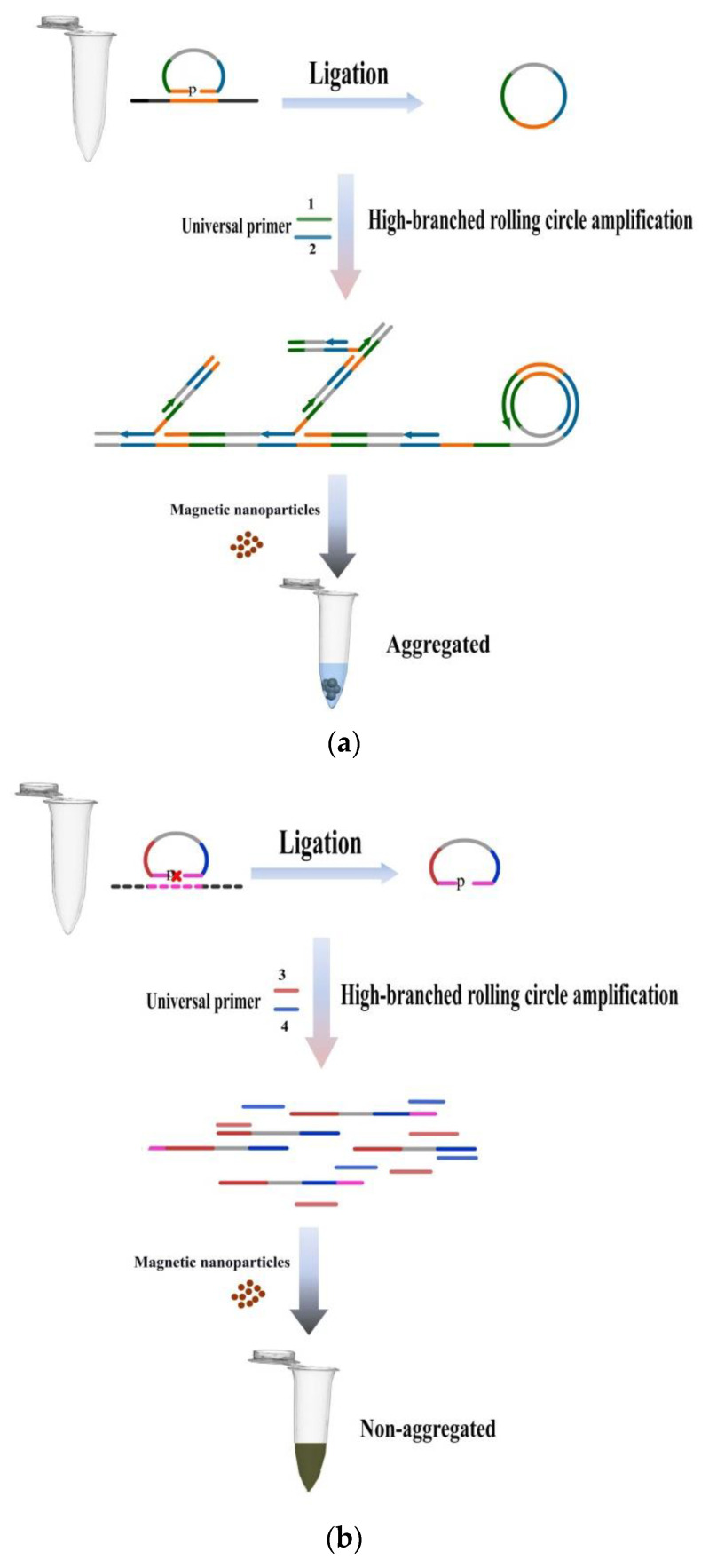

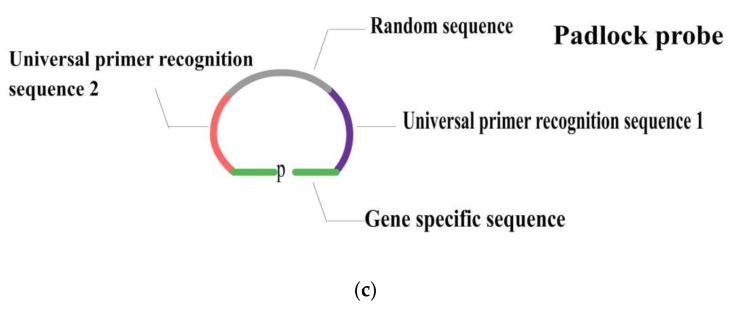
Schematic illustration of MHRCA-MPND. (**a**) When the target gene exists, the padlock probes is linked into a loop, and it is amplified with the universal primer by a hyperbranched rolling circle amplification. The product is irreversibly agglomerated with magnetic particles, and the detection result is visible to the naked eye. (**b**) When the target gene non-exists, the linking reaction and amplification reaction are not carried out, no HRCA product is entangled with the magnetic particles and the magnetic particles are re-suspended in the solution. (**c**) Padlock probe structure.

The principle of this assay is demonstrated in Figure 1. A padlock probe with a length of about 90 nucleotides consists of a target gene recognition sequence, a universal primer recognition sequence, a random sequence and a phosphorylated 5’ end (Figure 1c). First, all the padlock probes are connected in a tube. When target genes are present, the corresponding padlock probes form closed ring molecules; otherwise they will stay as linear probes. Then the product is distributed to different amplification reaction tubes, each tube containing a pair of universal primers to detect a specific target gene. Looped padlock probes and corresponding universal primers start hyperbranched rolling circle amplification (HRCA) under the action of polymerase, thus obtaining branched chain amplification products. The amplified products interact with the magnetic particles to form aggregated magnetic particle clusters, allowing the detection results to be visible to the naked eye (Figure 1a). On the contrary, no aggregated magnetic particle clusters are generated with linear probes, meaning no target gene was present and thus no bacteria was detected (Figure 1b).

HRCA increases the target sequence exponentially, and a large number of amplified products can be obtained in a short time during one experiment at a constant temperature. The application of magnetic particles makes the detection results visible to the naked eye. This research idea reduces the equipment relied on for temperature cycling reaction and detection signal processing, which greatly simplifies required equipment, allows automation and can be detected by the naked eye. This new method combined with a portable apparatus can be utilized in developing countries and under-resourced areas to allow quick and accurate visual detection on site.

### 2.1. Experimental Sample

All strains used in this study were purchased from the China Center of Industrial Culture Collection (CICC). Each of the bacteria strains was cultured following the manufacturer’s instruction, and a plate count was used to determine the stock concentration. The stock of each reference strain was heat lysed at 95 °C for 10 min. The reference strain and gene targets selected for each category are listed in Table 1. Table 2 shows the sequence information of probes and primers used in the experiment.

### 2.2. Ligation

A simulated target of each gene was designed to evaluate the feasibility of this method. The ligation mixture totaled 20 µL and contained 10 × Taq DNA Ligase Buffer 2 µL, 10 µM padlock probe 2 µL, Taq DNA Ligase 1 µL, 10 µM simulated target 2 µL or pre-heated reference strain sample; nuclease-free water was added to the reaction system to a total of 20 µL. The reaction mixture was incubated at 55 °C for 1 h.

### 2.3. HRCA Reaction

The HRCA was performed in a 40 μL volume containing 2 μL ligation product, 10 × Buffer 4 µL, 10 µM universal primer 2 µL, 10 mg/mL BSA 0.8 µL, 10 µM dNTP 1.6 µL, Phi29 DNA polymerase 1 µL and nuclease-free water to total the reaction system to 40 µL. The reaction mixture was incubated at 30 °C for 1 h.

### 2.4. Naked-Eye Detection

The magnetic particles and 50 ng HRCA reaction products were used for agglomeration experiments under the action of an external magnetic field. Whether HRCA products exist in the solution or not, the magnetic field will cause aggregation of magnetic particles. The steps of aggregation and resuspension are repeated 2–3 times by gently vortexing the reaction solution. The experimental results were observed by the naked eye. HRCA products and MPs are stretched, entangled and randomly woven with each other. They form aggregates under an external magnetic field. The strong physical adhesion and aggregation between HRCA products and MPs is irreversible and the magnetic beads are tightly bound together to prevent them from being re-suspended. Short-chain nucleic acids can easily disperse aggregates by several pipetting steps. The reason may be that short DNA strands are difficult to intertwine and form large visible coils. When HRCA product is present, the magnetic particles will agglomerate, and the solution is clean. When the HRCA product is not present, the magnetic particles will stay re-suspended, and the solution will be turbid.

### 2.5. Limit of Detection

To determine the specific concentration level at which the organisms could be correctly and consistently identified by the assay, titered bacterial pathogens were tested to evaluate the sensitivity. The estimate of the limit of detection was based on serial 10-fold dilution of each reference strain in molecular H_2_O range from 10^5^ to 1 CFU/mL; each dilution was tested with 10 replicates. The lowest concentration that has 100% performance was selected to determine the limit of detection. HRCA and visual inspection were carried out to observe whether the magnetic particles were resuspended, so as to determine the detection limit of each bacterial pathogen.

## 3. Results and Discussion

### 3.1. Amplification Reaction

**Figure 2:**
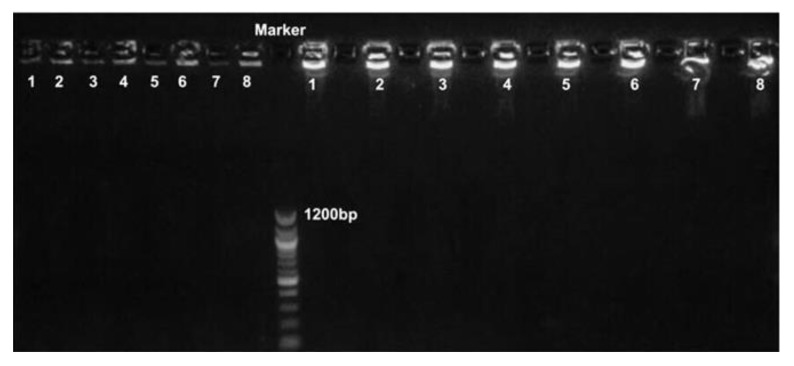
Gel electrophoresis of RCA reaction and HRCA reaction. In the middle of the gel electrophoresis map is a DNA Marker; the 8 wells left of the Marker are RCA products, and the 8 wells right of the Marker are HRCA products.

uidA gene was selected to use due to it being present in each reference strain. For both rolling circle amplification (RCA) reaction and HRCA, two different padlock probes were designed. Experiments were replicated a total of eight times to compare the results of amplification methods (Figure 2).

The final amplification products obtained by RCA and HRCA reactions are chains larger than 10 kb. When running these on an electrophoresis gel, the products are concentrated near the sample loading wells at the top of the gel. From the electrophoresis maps, it can be seen that the HRCA reaction produced more products than the RCA reaction.

RCA reaction products can only increase linearly. After one round of amplification, only one repeat sequence can be obtained, which greatly reduces the detection efficiency. The HRCA reaction introduced double primers, and the amplification of rolling circle and branched chain increased the target gene exponentially. Compared with RCA reaction, HRCA reaction obtains more products in the same time, improves the detection sensitivity and realizes large accumulation of products in a short time.

### 3.2. Magnetic Particles Selection

**Figure 3:**
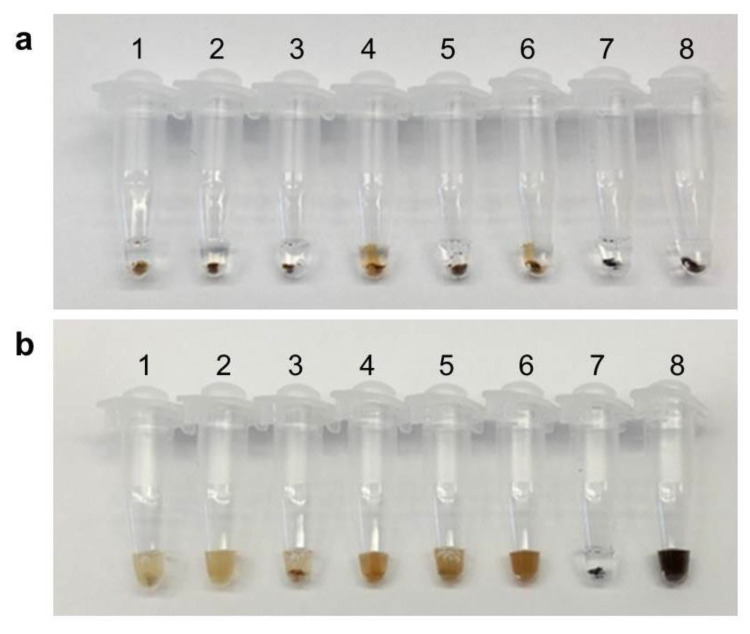
Agglomeration reaction of magnetic beads: 1~2, Dynal MyOne; 3~4, SPRI beads; 5~6, Magbio beads; 7~8, HMP; 2, 4, 6, 8 serve as negative control; (**a**)The agglomeration reaction before vortex; (**b**) The agglomeration reaction after vortex.

To determine the best magnetic bead for agglomeration, we compared the following beads: Dynal MyOne (Dynabeads MyOneTM Streptavidin C1, Invitrogen, Waltham, MA, USA), SPRI beads (Beckman Coulter, Brea, CA, USA), Magbio beads (Magbio, Gaithersburg, MD, USA) and home-made magnetic particles (HMP) [22]. The agglomeration reaction was carried out with 50 ng HRCA reaction product under the action of an external magnetic field. Agglomeration effects of the magnetic particles were observed before and after vortexing (Figure 3).

If HRCA products are present in the solution, magnetic beads will not resuspend in solution after vortexing and instead stay clumped together. If HRCA products are not present in the solution, the magnetic beads will resuspend easily, and the solution will be turbid. In the result shown in Figure 3b, all the negative controls showed very good resuspension after vortex. As for the positive samples 1, 3, 5 and 7, all four selected beads showed visible clumps after vortex, but obviously the HMP magnetic beads were the only type that still clumped tightly without any turbidity in the solution (Figure 3b); HRCA products tightly tie the magnetic beads together and have a strong physical attachment, preventing aggregation from being re-suspended. Therefore, HMP was chosen as the most suitable option for visual detection.

### 3.3. Magnetic Particles Amount

The number of magnetic particles is also an important factor affecting the experiment. If there are too many magnetic particles in the solution, they cannot entangle with amplification products and disperse in the solution, resulting in a waste of magnetic particles. In contrast, when there are too few magnetic particles, the aggregates are too small to observe the experimental results with the naked eye. All of the above can lead to misjudgment of the experimental results.

**Figure 4:**
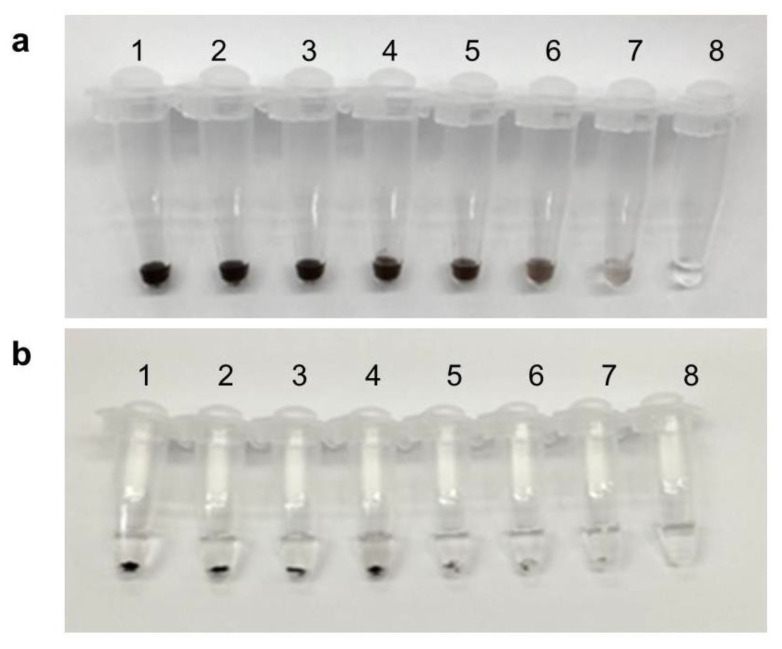
Optimization of magnetic beads input. (**a**) Before the agglomeration reaction; (**b**) After the agglomeration reaction. Numbers 1~8 represent the amount of magnetic beads added: 6000 ng, 4000 ng, 2000 ng, 1000 ng, 500 ng, 250 ng and 100 ng. Position 8 is a negative control.

To carry out agglomeration, we added 6000 ng, 4000 ng, 2000 ng, 1000 ng, 500 ng, 250 ng and 100 ng of HMP magnetic particles to both HRCA products and negative controls. Results were observed before and after agglomeration of magnetic particles (Figure 4).

### 3.4. Limits of Detection

**Figure 5:**
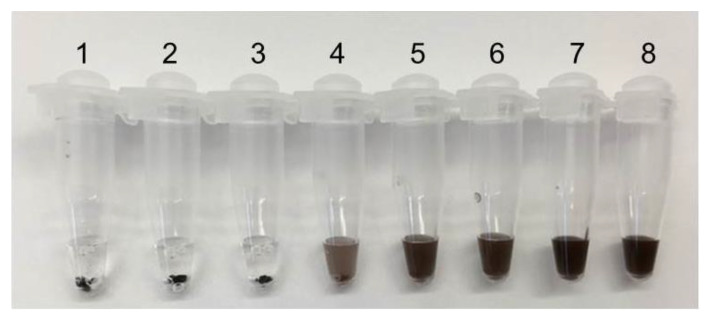
Limits of detection. Numbers 1~8 represent simulated probe concentrations of 1 µM, 100 nM, 10 nM, 1 nM, 100 pM, 10 pM, 1 pM and negative control.

Probes in the HRCA reaction were serial diluted starting at 1 µM and going down to 1 pM, with a negative control in the last position. The 8 HRCA products were agglomerated with 1000 ng of HMP to detect the sensitivity of the reaction. The results are shown in Figure 5.

In the higher concentration HRCA product solutions, magnetic particle aggregation clusters are tightly wound and present a visible magnetic particle aggregate in a tube (Figure 5, positions 1–3); in the low concentration solution of products, the magnetic particles cluster in a loose state (Figure 5, positions 4–7). When the probe concentration is 10 nM (Figure 5, position 3), the magnetic particles are tightly agglomerated with HRCA products. However, when the concentration of the product is below 1 nM (Figure 5, position 4), a large number of suspended magnetic particles stay in the solution. The results show that the limit of visual detection based on magnetic particles is 10 nM.

### 3.5. Food-Borne Pathogen Detection

**Figure 6:**
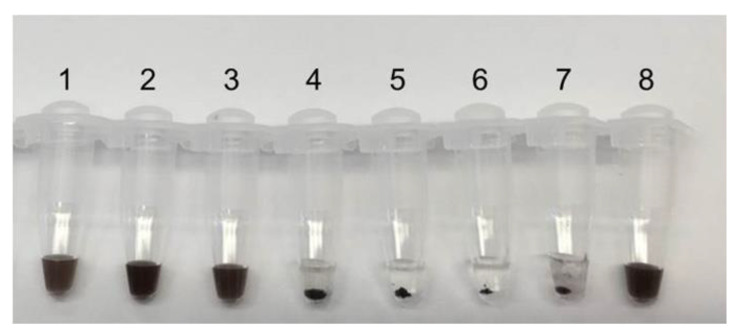
EHEC detection results. The concentration of EHEC is 1 × 10^3^ CFU/mL. 1–8: ST, LT, ipaH, eae, Stx1, rfbE, uidA gene and negative control, respectively.

We applied this method to detect five pathogenic microorganisms. After diluting each pathogenic bacteria to 10^5^, 10^4^, 10^3^, 10^2^, 10^1^ and 1 CFU/mL, the HRCA reaction and visual detection were performed to determine the detection sensitivity of each bacterium and each gene. Taking the detection result of EHEC at the concentration of 1 × 10^3^ CFU/mL as an example, the detection sensitivity of the method to pathogenic microorganisms was tested by agglomeration reaction with HMP; the result is shown in Figure 6. When the concentration of EHEC is 1 × 10^3^ CFU/mL, its amplification products form aggregates with magnetic particles, so the concentration can then be continuously reduced for experiments to determine the detection limit of EHEC.

For each strain, the visual detection is carried out at the same time for five different bacterial concentrations. The target genes corresponding to each strain are shown in Table 1, thus determining the detection limits of five food-borne pathogens (Table 3).

The detection limit of each strain is the maximum detection limit of the corresponding gene. The results show that the detection sensitivity of EHEC, ETEC, EPEC, EIEC and *E. coli* are 1 × 10^3^ CFU/mL, 1 × 10^4^ CFU/mL, 1 × 10^3^ CFU/mL, 1 × 10^4^ CFU/mL, and 1 × 10^2^ CFU/mL, respectively.

## 4. Conclusions

In conclusion, the naked-eye detection method of nucleic acids based on magnetic particles in HRCA greatly reduces the amount of equipment and reagents needed in the lab, thus reducing cost and trained professionals, allowing this user-friendly method to be used worldwide. HRCA technology allows a large amount of products to be accumulated in a short period of time. The amplicons in HRCA are wound with magnetic particles, and the detection results are visible to the naked eye. The method simplifies nucleic acid detection equipment and can achieve high throughput and automation. This method allows for a simple and low-cost nucleic acid detection technology to be developed for the detection of large-scale infectious diseases and genetic diseases and greatly reduces laboratory costs.

## Figures and Tables

**Table 1:** Specific target molecules corresponding to pathogenic bacteria.

Reference Strain	Strain	uidA	rfbe	stx1	eae	ipaH	LT	ST
*E. coli*	CICC 10305	+	-	-	-	-	-	-
EIEC	CICC 10661	+	-	-	-	+	-	-
EPEC	CICC 10664	+	-	-	+	-	-	-
ETEC	CICC 10667	+	-	-	-	-	+	+
EHEC	CICC 21530	+	+	+	+	-	-	-

**Table 2:** Probes and primers design.

Target Gene	Sequence Name	Sequence
uidA	Padlock probe	p-ATCACCATTCCCGGCGCGTCTCGTGGCTGAGAGCCTGGTAGTCGGAACTGGCAGGCAGGACGCACGCGTAAGCCGTTTTCATCGGTA
	Primer 1	CGTCTCGTGGCTGAGAG
	Primer 2	TTACGCGTGCGTCCTGC
	Simulate target	CGCCGGGAATGGTGATTACCGATGAAAACGGC
ST	Padlock probe	p-CATGCTTTCAGGACTACGCTCCATGATAGGCACGCCTGGTAGTCGGAACGTGGCAGGATTAGCTAGCCGATGTCCGCAGTAATTGCTACTATT
	Primer 1	GCTCCATGATAGGCACG
	Primer 2	GGACATCGGCTAGCTAATC
	Simulate target	GTAGTCCTGAAAGCATGAATAGTAGCAATTACTGC
LT	Padlock probe	p-GTTCCTCTCGCGTGATCTAACGGAGGCTAAGTTCCCTGGTAGTCGGAACGTGGCAGCGCAAGGCACCTCCTGCCAAAGCCGGTTTGT
	Primer 1	TAACGGAGGCTAAGTTC
	Primer 2	AGGAGGTGCCTTGCG
	Simulate target	GATCACGCGAGAGGAACACAAACCGGCTTTGGC
IpaH	Padlock probe	p-GGAAAGGCGGTCAAGGAACCACGTAAGGCCGTATCGACCTGGTAGTCGGAACGTGGCAGGTGTCAAGGCTTCACGCTGCTGGCAGAGACGGTATC
	Primer 1	CACGTAAGGCCGTATCGA
	Primer 2	GCGTGAAGCCTTGACAC
	Simulate target	GTTCCTTGACCGCCTTTCCGATACCGTCTCTGCCAGCA
eae	Padlock probe	p-CCGTTCCATAATGTTGTTGTGAAGCGTAGGCACCTGGTAGTCGGAACGTGGCAGAGTAAGGTCCTACCGGTCTGCAGATTAACCTCTG
	Primer 1	TTGTGAAGCGTAGGCA
	Primer 2	CCGGTAGGACCTTACT
	Simulate target	CAACATTATGGAACGGCAGAGGTTAATCTGCAGA
rfbE	Padlock probe	p-GTAATAGTTTTATTTCCAGAGCAGTCCTACCTTGCCCTGGTAGTCGGAACGTGGCAGAGGCAAGCGCGCATCTCCACCTTCACCTGTAG
	Primer 1	GAGCAGTCCTACCTTGC
	Primer 2	AGATGCGCGCTTGCCT
	Simulate target	TGGAAATAAAACTATTACTACAGGTGAAGGTGG
Stx1	Padlock probe	p-CAGACAATGTAACCGCTGCCAAGCCTGATCGTCTGCCTGGTAGTCGGAACGTGGCAGGTGTTCGAGCACGCTCCGTGGTATAGCTACTGTCAC
	Primer 1	CCAAGCCTGATCGTCTG
	Primer 2	GGAGCGTGCTCGAACAC
	Simulate target	CAGCGGTTACATTGTCTGGTGACAGTAGCTATACCAC
Negative Control	Padlock probe	p-GCTGACCTCGGCATGGACGCATGCCTTCTAACCCTGGTAGTCGGAACGTGGCAGCGCTTAGTAGCTCCACAGGTAGCACTATGCC
	Primer 1	ACGCATGCCTTCTAAC
	Primer 2	GTGGAGCTACTAAGCG
	Simulate target	CCATGCCGAGGTCAGCGGCATAGTGCTACCT

**Table 3:** Limit of detection for each Bacteria strain.

Strain	Gene	Detection Limit (CFU/mL)
EHEC		1000
	eae	1000
	uidA	1000
	rfbe	10
	stx1	100
ETEC		10,000
	LT	10,000
	ST	10,000
	uidA	1000
EPEC		1000
	eae	1000
	uidA	100
EIEC		10,000
	ipaH	10,000
	uidA	100
*E. coli*		100
	uidA	100

## Data Availability

Not applicable.

## References

[1] Riley L.W. (2020). Extraintestinal Foodborne Pathogens. Annu. Rev. Food Sci. Technol..

[2] Ishaq A.R., Manzoor M., Hussain A., Altaf J., Rehman S.U., Javed Z., Afzal I., Noor A., Noor F. (2021). Prospect of microbial food borne diseases in Pakistan: A review. Braz. J. Biol..

[3] Nelluri K.D.D., Thota N.S., Alexandru M.G., Alina M.H. (2018). Challenges in Emerging Food-Borne Diseases. Book Food Safety and Preservation.

[4] Matle I., Mbatha K.R., Madoroba E. (2020). A review of *Listeria monocytogenes* from meat and meat products: Epidemiology, virulence factors, antimicrobial resistance and diagnosis. Onderstepoort J. Vet. Res..

[5] Jokerst J.C., Adkins J.A., Bisha B., Mentele M.M., Goodridge L.D., Henry C.S. (2012). Development of a Paper-Based Analytical Device for Colorimetric Detection of Select Foodborne Pathogens. Anal. Chem..

[6] Rohr J.R., Barrett C.B., Civitello D.J., Craft M.E., Delius B., DeLeo G.A., Hudson P.J., Jouanard N., Nguyen K.H., Ostfeld R.S. (2019). Emerging human infectious diseases and the links to global food production. Nat. Sustain..

[7] Aboubakr H., Goyal S. (2019). Involvement of Egyptian Foods in Foodborne Viral Illnesses: The Burden on Public Health and Related Environmental Risk Factors: An Overview. Food. Environ. Virol..

[8] Hu J., Wang Y., Ding H., Jiang C., Geng Y., Sun X., Jing J., Gao H., Wang Z., Dong C. (2020). Recombinase polymerase amplification with polymer flocculation sedimentation for rapid detection of *Staphylococcus aureus* in food samples. Int. J. Food Microbiol..

[9] Raj V., Vijayan A.N., Joseph K. (2015). Cysteine capped gold nanoparticles for naked eye detection of *E. coli* bacteria in UTI patients. Sens. Bio-Sens. Res..

[10] Kwon D., Lee S., Ahn M.M., Kang I.S., Park K.-H., Jeon S. (2015). Colorimetric detection of pathogenic bacteria using platinum-coated magnetic nanoparticle clusters and magnetophoretic chromatography. Anal. Chim. Acta.

[11] Kim S.K., Cho H., Jeong J., Kwon J.N., Jung Y., Chung B.H. (2010). Label-free and naked eye detection of PNA/DNA hybridization using enhancement of gold nanoparticles. Chem. Commun..

[12] Lin C., Zhang Y., Zhou X., Yao B., Fang Q. (2013). Naked-eye detection of nucleic acids through rolling circle amplification and magnetic particle mediated aggregation. Biosens. Bioelectron..

[13] Safavieh M., Ahmed M.U., Sokullu E., Ng A., Braescuac L., Zourob M. (2014). A simple cassette as point-of-care diagnostic device for naked-eye colorimetric bacteria detection. Analyst.

[14] Ohk S.-H., Bhunia A.K. (2013). Multiplex fiber optic biosensor for detection of *Listeria monocytogenes, Escherichia coli O157:H7* and *Salmonella enterica* from ready-to-eat meat samples. Food Microbiol..

[15] Shi X., Yu L., Lin C., Li K., Chen J., Qin H. (2021). Biotin exposure-based immunomagnetic separation coupled with sodium dodecyl sulfate, propidium monoazide, and multiplex real-time PCR for rapid detection of viable *Salmonella typhimurium, Staphylococcus aureus,* and *Listeria monocytogenes* in milk. J. Dairy Sci..

[16] Maier C., Hofmann K., Huptas C., Scherer S., Wenning M., Luecking G. (2021). Simultaneous quantification of the most common and proteolytic *Pseudomonas* species in raw milk by multiplex qPCR. Appl. Microbiol. Biotechnol..

[17] Li F., Ye Q., Chen M., Shang Y., Zhang J., Ding Y., Xue L., Wu S., Wang J., Pang R. (2021). Real-time PCR identification of *Listeria monocytogenes* serotype 4c using primers for novel target genes obtained by comparative genomic analysis. LWT—Food Sci. Technol..

[18] Sun J., Shi Y., Du Y., Wang Z., Liu Z., Wang H., Zhao G., Ma Y., Zheng M. (2020). Rapid Detection of Diarrheagenic *Escherichia coli* by a New Multiplex Real-Time Quantitative PCR Assay. Appl. Biochem. Microbiol..

[19] Zhong Y., Wang Y., Zhao T., He X., Ke Y., Liu W., Zou D. (2020). Multiplex real-time SYBR Green I PCR assays for simultaneous detection of 15 common enteric pathogens in stool samples. Mol. Cell Probes.

[20] Suo B., He Y., Tu S.-I., Shi X. (2010). A Multiplex Real-Time Polymerase Chain Reaction for Simultaneous Detection of *Salmonella* spp., *Escherichia coli O157*, and *Listeria monocytogenes* in Meat Products. Foodborne Pathog. Dis..

[21] Zhao Y., Wang H., Zhang P., Sun C., Wang X., Wang X., Yang R., Wang C., Zhou L. (2016). Rapid multiplex detection of 10 foodborne pathogens with an up-converting phosphor technology-based 10-channel lateral flow assay. Sci. Rep..

[22] Li G., Shen B., He N., Ma C., Elingarami S., Li Z. (2011). Synthesis and Characterization of Fe_3_O_4_@SiO_2_ Core–Shell Magnetic Microspheres for Extraction of Genomic DNA from Human Whole Blood. J. Nanosci. Nanotechnol..

